# Gum Arabic containing *Allium sativum* L. essential oil-based nanoparticles as biofumigant grain protectant against *Callosobruchus maculatus* F.

**DOI:** 10.1371/journal.pone.0334926

**Published:** 2025-10-24

**Authors:** Huda H. Elbehery, Habiba A. Ahmed, Samar Sayed Ibrahim, Waleed Abouamer, Amr Farouk

**Affiliations:** 1 Pests and Plant Protection Department, National Research Centre, Giza, Egypt; 2 Plant Biochemistry Department, National Research Centre, Giza, Egypt; 3 Medical Biotechnology Department, Genetic Engineering and Biotechnology Research Institute, City of Scientific Research and Technological Applications, New Borg El- Arab City, Alexandria, Egypt; 4 Plant Protection Department, Faculty of Agriculture, Al-Azhar University, Cairo, Egypt; 5 Flavor and Aroma Chemistry Department, National Research Centre, Giza, Egypt; Ross Lifescience Limited, INDIA

## Abstract

Gum Arabic nanoparticles (GA NPs) were used to nano-encapsulate *Allium sativum* or garlic essential oil (GO) using the freeze-drying technique. The fumigant toxicity of GO and GO-GA nanoparticles was evaluated against *Callosobruchus maculatus*, a major pest of stored products. Adults were exposed to concentrations of 10.0, 5.0, 2.5, and 1.25 µL/L air for 24 hours to evaluate the lethal concentration (LC) values. Gas Chromatography-Mass Spectrometry for GO identified diallyl trisulfide (38.78%), allyl methyl trisulfide (23.93%), and diallyl disulfide (13.66%) as the main compounds. Dynamic light scattering and transmission electron microscope tests verified the stability and uniformity of the produced nanoparticles, which were distinguished by their small particle size (15.10 nm), low PDI value (0.31), and negative zeta potential (−10.20). A high encapsulation efficiency of 84.74 ± 1.74% was achieved for the produced nanoparticles. The linkage and interaction between GO and GA as a polymer were confirmed by Fourier transform infrared spectroscopy. After 24-hour exposure, GO-GA NPs resulted in lower LC_50_ values (1.14 µL/L air) than GO (2.08 µL/L air) against *C. maculatus* adults. The inclusion of GO-GA NPs at LC_40_ had a significant post-effect on progeny production of *C. maculatus*, resulting in a significant reduction in the number of deposited eggs and adult emergence, which led to a significant decrease in the percentage of adult emergence to 15.23 ± 5.46 compared to 61.33 ± 2.94, as observed in the GO treatment. GO-GA NPs enhanced the persistence activity, exhibiting a continued toxic effect for >30 days, with a PT_50_ of 22.29 days compared to 12.79 days for GO. This study suggested that nano-formulation could enhance the efficiency of garlic oil as an eco-friendly grain protectant to control *C. maculatus* adults.

## Introduction

Pulse beetles, *Callosobruchus maculatus* (F.) (Coleoptera, Chrysomelidae) are one of the most harmful primary pests of stored food products in tropical and subtropical regions, as well as being a worldwide pest of legume seeds [1]. During storage, beetles cause significant qualitative and quantitative damage to seeds, resulting in weight and germination loss [2]. In many developing countries, synthetic fumigants are widely used in storage facilities for managing insect pests [3,4]. However, the implementation of synthetic insecticides has been characterized by several drawbacks, including insect resistance, toxicity to humans and livestock, ozone depletion, and toxicity to non-target organisms [5]. Plant-based products, such as essential oils, can offer efficient and eco-friendly alternatives to chemical insecticides.

Essential oils are preferred for controlling insect pests due to their advantages, such as reduced environmental impact and low toxicity to humans [6,7]. Several essential oils and their phytoconstituents have been reported to possess repellency, oviposition deterrence, growth inhibitory, and insecticidal activity against different stored product insects, including *C. maculatus* [8–11]. It has been reported that the essential oil of garlic (*Allium sativum* L.) has antimicrobial, fungicidal, acaricidal, and insecticidal properties [12–15]. The insecticidal effectiveness of garlic oil and its components against stored product insects has been demonstrated in previous studies [16–18]. However, the poor bioavailability and low water solubility of essential oils prevent their widespread application in pest management [19]. Moreover, encapsulating essential oils and their constituents within protective shell walls is necessary due to their high susceptibility to the effects of external environmental factors such as light, temperature, and oxygen. The nanoencapsulation technique offers the long-term release of volatile substances, reduced toxicity to non-target organisms, and transformed physicochemical stability [20]. In addition to these advantages, essential oil-based bioinsecticides in nano-capsule form may increase toxicity and bioavailability, as well as fewer active ingredients might be needed [21]. Higher insecticidal efficacy was demonstrated by nano-encapsulated essential oils than by non-formulated oils [22,23].

Freeze-drying is a promising method for encapsulating bioactive substances. This method favors heat-sensitive materials, such as essential oils, and produces products of greater and longer-lasting quality [24]. Gum Arabic (GA) is widely used as a stabilizer, thickener, and emulsifier in the pharmaceutical, textile, cosmetics, and food industries [25]. Along with having low viscosity at high concentrations, gum Arabic exhibits excellent properties, including emulsification, the capacity to form films, and a small particle size [26]. Previous studies have documented the encapsulation of essential oils using gum Arabic [27–29]. Therefore, this work aimed to encapsulate garlic oil by a freeze-drying technique using GA. The physicochemical characteristics of the produced nano-formulation were examined, including Z-average (d.nm), polydispersity index (PDI), zeta potential, and encapsulation efficiency. Garlic oil (GO) has been tested against *C. maculatus* adults for its fumigant toxicity and persistence activity in pure and nano-encapsulated forms.

## Results and discussion

### Volatile identification of garlic essential oil (GO)

**Fig 1:**
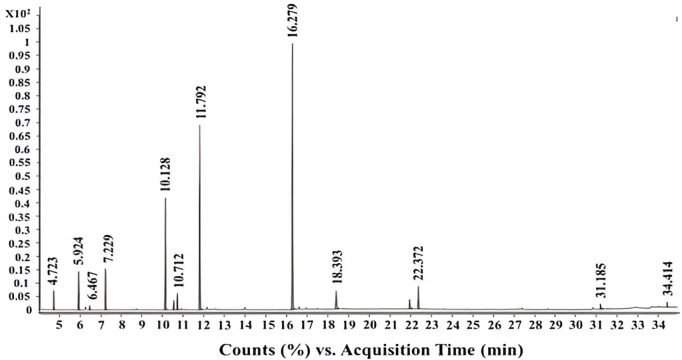
GC-MS chromatogram of garlic essential oil.

GO composition was identified using Gas Chromatography-Mass Spectrometry (GC-MS) analysis (Table 1 and Fig 1). The data illustrated that fourteen different compounds were identified. The major sulfur-containing compounds were diallyl trisulfide (38%), allyl methyl trisulfide (23%), and diallyl disulfide (13%). Among the other sulfur-containing substances were dimethyl trisulfide (4.58%), allyl methyl disulfide (3.88%), diallyl tetrasulfide (2.95%), allyl (*Z*)-1-propenyl disulfide (2.01%), allyl (E)-1-Propenyl disulfide (1.28%), and methyl *trans*-1-propenyl disulfide (0.46%). The oil composition exhibits quantitative variations compared to previous studies [30,31]. The essential oil constituents vary in quantity and quality depending on climate and location. It was reported that diallyl disulfide (25.2%), allyl methyl trisulfide (23.8%), and diallyl trisulfide (21.1%) were detected in an Egyptian garlic essential oil as the major constituents [32].

**Table 1:** Volatile constituents of Garlic essential oil identified by GC-MS.

Peak	RT^a^	Compound	RI^b^	Area^c^ %
1	4.72	Diallyl sulfide	861	1.77
2	5.92	Allyl methyl disulfide	920	3.88
3	6.47	Methyl trans-1-propenyl disulfide	940	0.46
4	7.23	Dimethyl trisulfide	970	4.58
5	10.13	Diallyl disulfide	1081	13.66
6	10.54	Allyl (E)-1-Propenyl disulfide	1103	1.28
7	10.71	Allyl (Z)-1-Propenyl disulfide	1107	2.01
8	11.79	Allyl methyl trisulfide	1142	23.93
9	16.28	Diallyl trisulfide	1297	38.78
10	18.39	Methyl cinnamate	1380	3.21
11	21.95	Ethyl 4-ethoxybenzoate	1522	1.81
12	22.37	Diallyl tetrasulphide	1532	2.95
13	31.19	Palmitic acid	1968	1.09
14	34.41	Heptacosane	2700	0.59
–	–	Total	–	100

RT^a^: Retention time (min). RI^b^: Retention index measured on an HP-5ms column based on a homologous series of n-alkanes. Area Sum^c^: Peak area/Total peak area.

### Physicochemical properties of nanoparticles

The study aimed to improve the efficacy of GO against *C. maculatus* by encapsulating it in GA nanoparticles. Nano-encapsulation has gained popularity due to its advantages over bulk substances. Lyophilization stabilizes heat-sensitive substances, such as essential oils [33]. GA is a favored encapsulation material due to its low cost, non-toxicity, biocompatibility, and water solubility [34–36]. It effectively traps compounds during dehydration, minimizing volatile loss and exposure to air.

**Fig 2:**
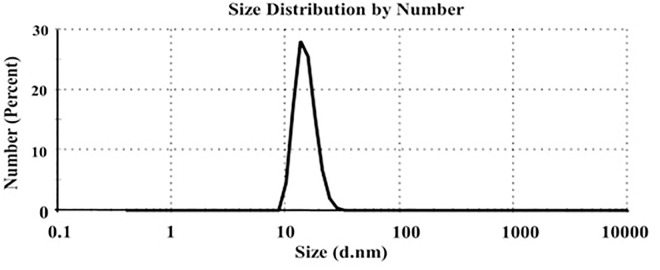
Particle size distribution of GO-GA NPs.

Table 2 and Fig 2 indicate the variables influencing the stability of nanoparticles: zeta potential, particle size, and polydispersity index (PDI). Particles had a negative surface charge (zeta potential of −10 mV), and their dimension was in the nanoscale (15 nm). Furthermore, the polydispersity index value was 0.31. The parameters of DLS measurements significantly influence the physical stability and homogeneity of the produced nano-capsules. Data demonstrates that the nano-capsules had a low PDI value below 0.50, indicating a good distribution of the particles [37]. Zeta potential measures a nanoparticle’s surface characteristics. Highly negative or positive zeta potential values result in more stable formulations than those with low values [38]. In a previous study, different formulations of jasmine essential oil-gelatin/gum Arabic complex were synthesized with particle size, PDI, and zeta potential ranges from 74.58 to 384.14 nm, 0.16 to 0.66, and −8.67 to −1.92 mV, respectively [39].

**Table 2:** Particle size distribution, PDI, and zeta potential of GO-GA NPs.

Index	Value
Particle size (nm)	15.10
PDI	0.31
Zeta potential (mV)	− 10.20
EE%	84.74 ± 1.74%

**Fig 3:**
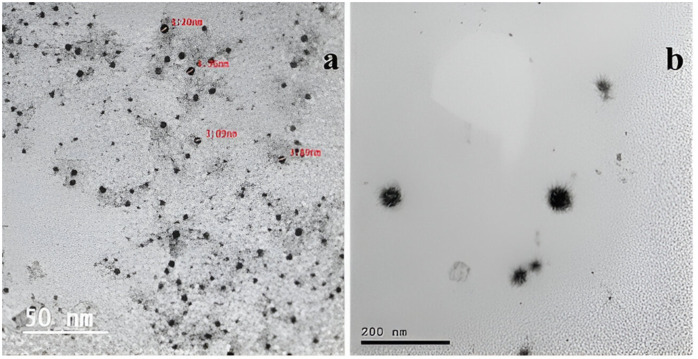
Transmission electron micrographs of GA NPs (a) and GO-GA NPs (b).

The morphology of GA and GO-GA NPs was analyzed using a TEM. Both GA (Fig 3a) and GO-GA NPs (Fig 3b) demonstrated a spherical shape, good dispersion, and narrow size distribution, confirming the efficacy of the nanoencapsulation process. The TEM and dynamic light scattering results differed slightly, with TEM yielding lower values due to possible shrinkage during drying, as noted by Rajabi et al. [40], who reported TEM sizes for saffron encapsulated by GA-Chitosan between 123–127 nm, compared to DLS sizes of 183–193 nm. The GO-GA NPs had a larger average diameter than GA NPs, potentially due to Ostwald ripening, where smaller droplets transfer to larger ones in the emulsion system [41].

The produced GO-GA NPs had an encapsulation efficiency percentage (EE%) of 84.74 ± 1.74%, as indicated in Table 2. The EE% is a crucial parameter because a high essential oil content should be retained throughout the encapsulation process [42]. Our study demonstrates that GA can effectively preserve the volatile components of GO, resulting in enhanced bioavailability and insecticidal activity. Similarly, the encapsulation of basil oil using GA resulted in an EE% ranging from 56 to 91% [43]. In a previous study, the essential oil of *Cymbopogon citratus* encapsulated in modified and unmodified gum Arabic yielded EE% values of 97 and 85%, respectively [28]. Additionally, it was reported that orange oil encapsulated in gum Arabic had EE% 75.9% [44].

**Fig 4:**
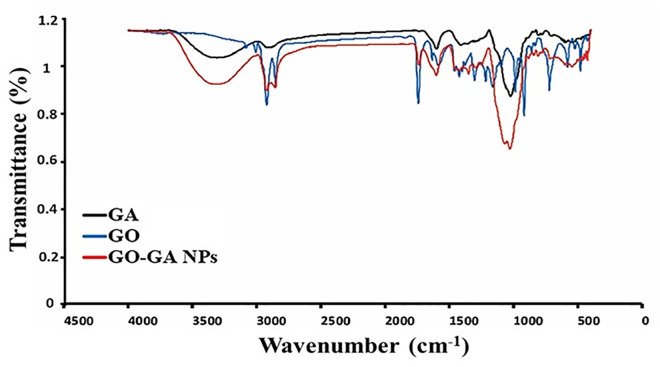
Fourier-transform infrared spectroscopy (FTIR) spectra of GA (black), GO (blue), and GO–GA NPs (red).

The association level between garlic essential oil and gum Arabic as a carrier in nano-preparation is shown in the FTIR spectrum illustrated in Fig 4. The spectrum of GA (in black) contains strong bands at 3339, 2881, 1607, and 1404 cm^−1^, which are assigned to the -OH, -CH_2_ group, and asymmetric and symmetric stretching vibration of the carboxylic acid salt -COO, respectively. The 1062 and 610 cm^−1^ peaks are assigned to the stretching vibrations of the C-O and -C-H bonds, respectively [45]. The peaks in the GO (in blue) at 1634 cm^−1^ are related to the -C = C- bond. The absorptions between 1400 and 1300 cm^−1^ are associated with the asymmetric angular deformation of the = CH_2_, rocking vibration of the -C-H bonds, and symmetric deformation of the -CH_3_ groups. The bands from 1250 to 1100 cm^-1^ are assigned to deformations of methylene and =CH_2_ of the vinyl group; additionally, the high-intensity peaks in the region between 990 and 900 cm^−1^ refer to the = CH_2_ deformation of the vinyl group present in the sulfides and vinyl dithiiins, the significant compounds of GO formed by the decomposition of allicin [46]. The spectra of the GO-GA NPs exhibited a similar trend to that of separate GO and GA (Fig 4, in red). The intensity of the peaks in the composite was reduced compared to those of the separate GO and GA, indicating the interaction between the composite components. For example, the shifting to a lower region in the composite at 1607−1634 and 900−990 cm^-1^, compared to the separate GA and GO.

### Insecticidal effect of GO and GO-GA NPs

**Fig 5:**
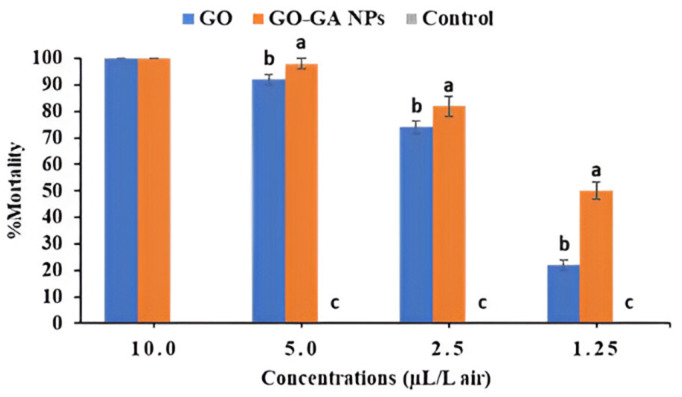
Mortality of *C. maculatus* after 24h exposure to GO and GO-GA NPs. Mean (±SE) values with different letters within the same concentration are significantly different, *P* <0.05, ANOVA, Duncan test.

Data in Fig 5 illustrate the fumigant toxicity effect of GO and GO-GA NPs against *C. maculatus* adults after 24h of exposure. The mortality rate was observed to be directly proportional to the increase in the tested concentration of both GO and GO-GA NPs (S1 Table). Fumigation with 10 µL/L air of both treatments resulted in 100% adult mortality compared to the control group (0.0% mortality). However, GO-GA NPs significantly increased the percent mortality when tested at concentrations of 5.0, 2.5, and 1.25 µL/L in air. Accordingly, the calculated LC values were lower due to GO-GA NPs treatment than those of GO treatment. As shown in Table 3, the LC_40_, LC_50_, and LC_90_ were 0.70, 1.14, and 3.34 µL/L air after GO-GA NPs treatment, compared to 1.64, 2.08, and 4.28 µL/L air, respectively, obtained after GO treatment.

**Table 3:** Toxicity of GO and GO-GA NPs on *C. maculatus* adults after 24h exposure.

Fumigant	LC_40_ (Confidence Limits µL/L air)	LC_50_ (Confidence Limits µL/L air)	LC_90_ (Confidence Limits µL/L air)	Slope±SE	Chi^2^
GO	1.64 (1.06- 2.06)	2.08 (1.60- 2.48)	4.28 (3.71- 5.26)	0.5815 ± 0.09	10.53
GO-GA NPs	0.70 (−0.39- 1.24)	1.14 (0.29-1.60)	3.34 (2.80-4.44)	0.5819 ± 0.12	9.72

Several studies have examined the potential application of essential oils and their constituents as fumigants against stored product insect pests [11,47–49]. According to the current study, the lower LC values obtained by nano-encapsulated GO indicate higher toxicity against *C. maculatus* adults. Our results agreed with Khoobdel et al. [50], who reported significant fumigant toxicity of nano-capsulated rosemary essential oil on *T. castaneum* compared to free oil. The fumigant toxicity of GO may be due to its volatile constituents. Sulfur compounds are thought to be responsible for the insecticidal effects of GO.

Our results are in line with those of other researchers who have examined the effectiveness of GO against stored-product insects and related its toxic effect to the main components, such as diallyl disulfide [17,51,52]. Additionally, according to Demeter et al. [18], GO exhibited the highest toxicity against *S. granaries* among the 25 essential oils tested. It was found that nano-formulation of *Cuminum cyminum* oil exhibited higher fumigant toxicity with a lower LC_50_ value (16.25 μL/L) than free oil (32.12 μL/L) against *T. castaneum* [8]. A remarkable increase in the toxicity of geranium and bergamot oils by their nano-formulations was observed against *T. castaneum* and *R. dominica* [47]. Our findings revealed that GA NPs enhanced the fumigant toxicity of GO. The higher volatility of an essential oil causes it to break down more quickly, which lowers its bioavailability and insecticidal effect [19].

On the other hand, increased efficacy comes from controlling the release of the essential components and protecting them through encapsulation [21]. GA functions efficiently as an encapsulating material for unstable substances during freeze-drying [53]. The enhanced toxicity of encapsulated oil may be due to the well-dispersed GO in the wall material, increased surface area, and good penetration [50].

### Post effect on progeny production

Compared to free GO, GO-GA NPs significantly reduced progeny production and adult emergence of *C. maculatus* in the present study. As illustrated in Table 4, GO-GA NPs significantly reduced the mean number of deposited eggs to 3.46 compared to 15.73 and 59.93 eggs resulting from GO treatment and control, respectively. Accordingly, the average number of emerging adults drastically decreased because of GO-GA NPs treatment, resulting in only 15.23% of adults appearing, compared to 61.33% for GO treatment. Therefore, insects treated with GO-GA NPs had a much higher percentage of inhibition rate than insects treated with non-formulated oil.

**Table 4:** Post-effect of GO and GO-GA NPs on progeny production of *Callosobruchus maculatus.*

Fumigant	The mean number of eggs	The mean number of emerged adults	The mean of % Emergence	%IR^a^
GO	15.73 ± 0.68b	9.66 ± 0.63b	61.33 ± 2.94b	36.80
GO-GA NPs	3.46 ± 0.46c	0.80 ± 0.29c	15.23 ± 5.46c	84.30
Control	59.93 ± 0.85a	58.20 ± 1.07a	97.05 ± 0.76a	–
F value	1854.882^*^	1737.858^*^	128.782^*^	–

Mean (±SE) values with different letters within the same column are significantly different, *P* <0.05, ANOVA, Duncan test.

* Highly significant. ^a^The inhibition rate (IR) is represented in (%).

The prepared nano-formulation in the current study may accelerate the diffusion of oil volatiles through the cuticle of insects, which might be responsible for the increased fumigant toxicity of GO-GA NPs. Furthermore, the mean number of deposited eggs and the progeny production of *C. maculatus* treated with GO-GA NPs decreased significantly. Our results agreed with earlier findings that clove essential oil loaded into PEG nanoparticles significantly reduced the progeny production of *R. dominica* compared to insects treated with pure oil [54]. Earlier studies reported reduced eggs and/or C. maculatus females using the LC_50_ of *Vanillosmopsis arborea* essential oil and its component α-bisabolol [55]. They explained their results by the raised sensitivity of mated insects to monoterpenoids. Additionally, lack of respiratory activity, accumulation of toxic metabolites, and the barrier effect of the essential oil may all contribute to reduced fecundity and egg mortality [56]. The continuous and stable release may indicate the enhanced insecticidal toxicity and physical stability of encapsulated essential oil [57]. According to the current study, GA nanoparticles preserved the volatile components of GO during the experiment, which explains the significant post-effect on *C. maculatus* progeny production.

### GO and GO-GA NPs persistence activity

**Fig 6:**
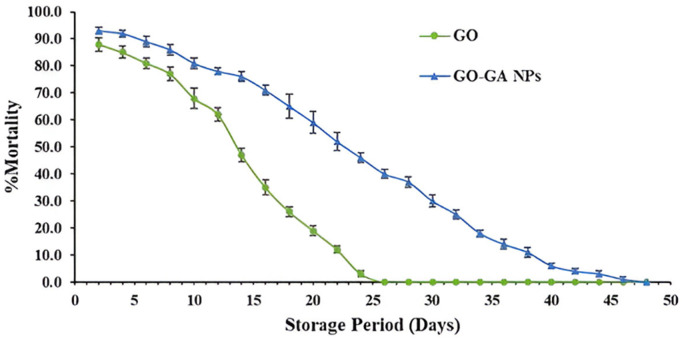
Mean persistence (±SE) of GO and GO-GA NPs on *Callosobruchus maculatus.*

Data presented in Fig 6 shows the persistent activity of GO and GO-GA NPs. GO-GA NPs significantly increased the persistence effect, causing LC_90_ to result in over 50% adult mortality even after 22 days of storage (t: −11.094; P < 0.0001). Only 12% of the insects died during the same storage period due to GO. After 24 days of storage, non-formulated GO lost its toxic effects, whereas GO-GA NPs caused 46% adult mortality (t: −19.230; P < 0.0001). The nano-encapsulated formulation prolonged the release of GO over time and caused 30% insect mortality even after 30 days (t: −13.416; P < 0.0001), as shown in S2 Table. The half-life times (PT_50_) for GO and GO-GA NPs were 12.79 and 22.29 days, respectively (Table 5), indicating the higher persistence of nano-encapsulated GO compared to free oil.

**Table 5:** PT_50_ values of GO and GO-GA NPs against *Callosobruchus maculatus.*

Fumigant	PT_50_ (Days)	95% confidence limits (Days)		Slope±SE	Chi^2^
		Lower	Upper		
GO	12.79	12.21	13.36	−0.14 ± 0.006	20.284
GO-GA NPs	22.29	21.53	23.06	−0.80 ± 0.003	9.298

In the present work, GO-GA NPs significantly boosted the persistence effect against *C. maculatus* adults. The persistence study demonstrated the increased half-life of GO-GA NPs, indicating their stability and slow release, which leads to efficient management of *C. maculatus*. On the other hand, a reduced half-life pesticide will only be effective for a short period. It must be applied multiple times at higher concentrations, which increases the risk of potential effects on non-target organisms and raises application costs [58]. Our results are consistent with a previous study, where the nano-formulation of *Cuminum cyminum* essential oil lost 15% and 60% of its toxic action against *T. confusum* and *S. granaries*, respectively, after 12 days, whereas the free oil lost all of its fumigant toxicity completely after the same exposure period [57]. They reported that the PT_50_ of *C. cyminum* and nano formulation was 1.62 and 9.05 days for *S. granarius*; and 2.25 and 21.21 days for *T. confusum*, respectively.

Additionally, when tested against *Ephestia kuehniella*, *Mentha longifolia* essential oil nano-formulation demonstrated slow release and increased stability with PT_50 _= 17.13 days compared to 2.39 days for free oil [59]. The toxic effect of essential oils on insects is due to their penetration into the insect’s body and respiratory system. However, utilizing GA as a wall material to increase the hydrophilicity of garlic oil allows for better oil delivery to the target site in insects. The persistent toxicity of nanoparticles may be caused by the controlled release of GO constituents from nano-formulation, which allows the slow release of small quantities over longer times.

## Materials and methods

### Chemicals and plant materials

Garlic essential oil (Allium sativum) was purchased from the Medicinal Plants and Extracts Unit, National Research Centre (NRC) in Cairo, Egypt. Tween 80, gum Arabic, and HPLC grade solvents were purchased from Sigma-Aldrich. Infestation-free cowpea seeds [Vigna unguiculata (L.) Walp.] were purchased from the local market in Cairo, Egypt, and were used for insect rearing and experimentation.

### Test insect

The insecticide-free stock culture of *C. maculatus* was established in the Department of Pests and Plant Protection at the NRC for several generations. Insects were allowed to feed on cowpea seeds in glass jars (1000 mL) covered with muslin cloth for ventilation. Rearing insects and experiments were performed under laboratory conditions (30 ± 2 °C and 65 ± 5% RH). Adult insects 2–3 days old were used for the experiments.

### GC-MS analysis of garlic oil

GC-MS analysis was performed using an Agilent 8890 GC System, connected to an Agilent 5977B GC/MSD mass spectrometer, to identify the structure of garlic essential oil. Helium was used as the carrier gas at a flow rate of 1 mL/min and a fused silica capillary column (30 m, 0.25 mm internal diameter, 0.25 mm film thickness). Furthermore, the temperature program was initially fixed at 45°C for 3 minutes, then increased to 150°C at a rate of 3°C/min, and from 150°C to 200°C at a rate of 5°C/min. 1 µL of the essential oil was injected into the GC at 230°C with a split ratio of 1:50. Mass spectra in electron EI mode (EI) at 70 eV were obtained, with *m*/*z* ranging from 39 to 500 amu. Peaks were identified by comparing them to NIST standards and published data. Percentages of detected compounds were calculated using GC peak areas. The retention index of each compound was determined using retention times of C_6_–C_26_ n-alkanes and compared to literature values [60].

### Preparation of garlic oil-gum Arabic nanoparticles inclusion

The encapsulation of garlic oil (GO) using gum Arabic (GA) nanoparticles was performed through an emulsification process followed by lyophilization, as described by Kaushik and Roos [53], with some modifications. In brief, GA was dissolved in deionized water for 60 min at 40 ºC to obtain a 20% (w/w) concentration of GA. The solution was cooled to room temperature, and garlic oil dispersed in Tween 80 (1:1) was added to obtain a 2% oil concentration [47]. The former mixture was agitated in a shaker at 25°C for 72h before being sonicated for 5 minutes using Daigger ultra-sonic (Model GEX 750, USA) to have a stable emulsion, followed by freezing for 20h at −20 ºC. The frozen emulsion was then subjected to lyophilization at −55°C for 24h. The resulting lyophilized substance was ground, sifted, and stored in an airtight container at −20°C until it was used. The powder was redispersed in deionized water (2.4 g/10 mL) for insecticidal bioassays.

### Characterization of GO-GA NPs inclusion

#### Particle size distribution.

After preparing GO-GA NPs, dynamic light scattering (DLS) was used to determine the particle size distribution, polydispersity index (PDI), and zeta potential values (NanoPartica SZ-100 apparatus, equipped with a 514 nm laser, Horiba Scientific). A quantity of 0.2 g was dissolved in distilled water (10 mL). The suspension was allowed to equilibrate for 30 minutes at room temperature to ensure the NPs were thoroughly dispersed and stable. The previous suspension was filtered using filter paper (Whatman NO.1) to eliminate any aggregates or large particles that might affect the measurement [61].

#### Transmission electron microscope (TEM).

The morphology of the prepared GO-GA NPs was photographed using TEM (JEM 2100 HRT, High-Resolution, Japan). A quantified sample of the prepared nanoparticles was dissolved in distilled water and then homogenized by a 10-minute sonication process. The solution was then dropped onto a carbon-coated copper grid. The sample was treated with 2% phosphotungstic acid and allowed to dry for 10 minutes at 28°C before examination [62].

#### Encapsulation efficiency (EE%).

The encapsulated GO using GA was dissolved in ethanol and placed in an ultrasonic water bath. The solution was then centrifuged and filtered [63]. A UV-Vis spectrophotometer (T80 + UV/VIS Spectrophotometer, PG Instruments Ltd.) was used to measure absorbance at 220 nm in triplicate. A calibration curve was developed using ethanol solutions with GO concentrations ranging from 0.05 to 1.25 mg/mL. The following equation (1) was used to calculate concentrations:


A = 0.0623 C + 0.0517 (1)

A: Absorbance, C: Concentration


EE% was calculated using the equation (2): EE%= (A/B) X 100 (2)

A: The content of GO in GA inclusion, B: The initial amount of GO used to prepare nanoparticles.

#### FTIR.

The association level between GO and GA in nanocapsules was evaluated using Fourier transform infrared spectroscopy (FTIR, Vertex 80v, Bruker, Germany), with a wavenumber range of 4000−400 cm^-1^ and a resolution of 4 cm^-1^.

### Insecticidal toxicity

The fumigant toxicity of GO and GO-GA NPs on *C. maculatus* adults was examined using the filter paper dip technique. Separate Whatman No. 1 filter paper discs (2 cm in diameter) were impregnated with different concentrations (10.0, 5.0, 2.5, and 1.25 µL/L air) of free oil and nano-capsules (containing 2% GO). Following that, treated filter paper discs were attached to the underside of the screw caps of 1000 mL glass jars. The cap was tightly screwed on after ten adult insects and 40 g of cowpea seeds were placed inside the jar. Five replications of each treatment and control were established. All groups were observed 24h after exposure, and the mortality of insects was recorded when legs and antennae showed no movement. A preliminary experiment using a maximum concentration (10.0 µL/L air) of GA solution alone was conducted to evaluate the possible toxic effect of GA on *C. maculatus* adults. Gum Arabic treatment showed no toxic effect on insects for the same exposure period (24h) as the negative control.

### Fecundity and progeny production

Sublethal concentration (LC_40_) obtained from the previous experiment was used to examine the post-effect of GO and GO-GA NPs on fecundity (number of eggs/insect female) and number of emerged adults. LC_40_ was chosen to ensure sufficient adult survival for evaluating sublethal effects on progeny production, as higher mortality at higher LC values could interfere with reproductive assessment. In a glass jar filled with 40 g of cowpea seeds, a one-sexed pair of *C. maculatus* adults was released separately and fumigated using the abovementioned method. The group that received no oil fumigation was maintained as a control. Fifteen replicates were used in the experiment. Daily, the number of eggs laid by each female on the seeds was counted in both the treated and untreated groups. The following calculation (3) was used to determine the mean percentage of adult emergence:


%Em= (NA/NE) X 100 (3)

%Em: % of Emergence, NA: Number of emerged adults, NE: Number of eggs laid

The inhibition rate percentage (%IR) was calculated as shown in equation (4) [64]:


%IR= (NC−NT)/NC X 100 (4)

%IR: percentage of inhibition rate, NC: Mean number of individuals in the untreated group, NT: Mean number of individuals in the treated group

### Persistence activity

The persistence activity of GO and GO-GA NPs was investigated using a high lethal concentration of LC_90_, as it ensured a strong initial mortality level suitable for monitoring residual toxicity over time after a single exposure. The tested concentration was utilized with the filter paper dip technique described above. Twenty *C. maculatus* adults were introduced to each jar every two days, with five replications for each treatment. Insect mortality was recorded after 48 hours. The experiment was carried out until the tested oils lost their ability to kill insects.

### Statistical analysis

Data from fumigation toxicity, fecundity, and progeny production were analyzed using a One-Way analysis of variance (ANOVA), and a probability of 0.05 was used in the Duncan test to differentiate between means. An independent samples t-test was performed to compare the data of the treated groups with GO and GO-GA NPs in the persistence experiment. The lethal concentration (LC) and 50% persistent time (PT_50_) values were determined by Regression using Finney’s probit analysis [65]. The entire dataset was processed using SPSS version 20.0.

## Conclusions

The current study enhanced the fumigant toxicity of GO using an encapsulation system with GA nanoparticles. The produced nano-formulation is characterized by small particle size and low PDI values, which show that it is more stable and has better dispersion. Moreover, high encapsulation efficiency for the synthesized nano-capsules (>80%) indicates that a sufficient amount of GO was successfully encapsulated in GA. GA nanoparticles enhanced the fumigant and residual toxicity of GO against *C. maculatus* adults. Compared to free GO, the nanoparticles had a significant impact on the progeny production of the tested insects. Nanoparticles of GO-GA inclusion exhibited a higher inhibition rate compared to free GO. LC90 of GO-GA NPs also showed a significantly higher persistence effect on *C. maculatus*. This study suggested that GO-GA NPs could lead to the long-term and widespread use of GO as a green protectant to control adults of *C. maculatus*. Large-scale applications of GO could be developed by employing GA to produce encapsulated oil, where small quantities are required and application times are reduced. However, further research is needed to assess the stability of nanoparticles under varying temperatures and moisture conditions, as well as their effects on both target and non-target organisms.

## Supporting information

S1 Table Mortality of *C. maculatus* after 24h exposure to GO and GO-GA NPs. (DOCX)

S2 Table Mean persistence (±SE) of GO and GO-GA NPs on *Callosobruchus maculatus.* (DOCX)
