# Supplementary material for: Gum Arabic containing Allium sativum L. essential oil-based nanoparticles as biofumigant grain protectant against Callosobruchus maculatus F
Source: PLoS One. 2025 Oct 24;20(10):e0334926. doi: 10.1371/journal.pone.0334926 (PMC12551849; doi:10.1371/journal.pone.0334926)
Supplement: S1 Table — (DOCX) [file pone.0334926.s001.docx]

**Table S1.** Mortality of *C. maculatus* after 24h exposure to GO and GO-GA NPs.

|  | Concentration (µL/L air) | | | |
| --- | --- | --- | --- | --- |
|  | 10 | 5 | 2.5 | 1.25 |
| GO | 100±0.00 | 92.0±2.0b | 74.0±2.44b | 22.0±2.0 |
| GO-GA NPs | 100±0.00 | 98.0±2.0a | 82.0±3.74a | 50.0±3.16 |
| Control | 0.00±0.00 | 0.00±0.00c | 0.00±0.00c | 0.00±0.00 |
| F(df), *p* value | - | 1131.5 (2), *p*<0.001 | 306.6 (2), *p*<0.001 | 134.57 (2),*p*<0.001 |

Mean (±SE) values with different letters within the same column are significantly different, *p* ˂0.05, ANOVA, Duncan test.
