# Supplementary material for: Gum Arabic containing Allium sativum L. essential oil-based nanoparticles as biofumigant grain protectant against Callosobruchus maculatus F
Source: PLoS One. 2025 Oct 24;20(10):e0334926. doi: 10.1371/journal.pone.0334926 (PMC12551849; doi:10.1371/journal.pone.0334926)
Supplement: S2 Table — (DOCX) [file pone.0334926.s002.docx]

**Table S2**: Mean persistence (±SE) of GO and GO-GA NPs on *Callosobruchus maculatus*

| Storage period (days) | GO | GO-GA NPs | *t^*^*(df), *p* value |
| --- | --- | --- | --- |
| 2 | 88.00±2.55 | 93.00±1.22 | -1.76 (8), *p*=0.11 |
| 4 | 85.00±2.24 | 92.00±1.22 | -2.74 (8), *p*=0.02 |
| 6 | 81.00±1.87 | 89.00±1.87 | -3.02 (8), *p*=0.01 |
| 8 | 77.00±2.55 | 86.00±1.87 | -2.84 (8), *p*=0.02 |
| 10 | 68.00±3.74 | 81.00±1.87 | -3.10 (8), *p*=0.01 |
| 12 | 62.00±2.55 | 78.00±1.22 | -5.65 (8), *p*<0.0001 |
| 14 | 47.00±2.55 | 76.00±1.87 | -9.17 (8), *p*<0.0001 |
| 16 | 35.00±2.74 | 71.00±1.87 | -10.85 (8), *p*<0.0001 |
| 18 | 26.00±1.87 | 65.00±4.47 | -8.04 (8), *p*<0.0001 |
| 20 | 19.00±1.87 | 59.00±4.00 | -9.05 (8), *p*<0.0001 |
| 22 | 12.00±1.22 | 52.00±3.39 | -11.09 (8), *p*<0.0001 |
| 24 | 3.00±1.22 | 46.00±1.87 | -19.23 (8), *p*<0.0001 |
| 26 | 0.00±0.00 | 40.00±1.58 | -25.29 (8), *p*<0.0001 |
| 28 | 0.00±0.00 | 37.00±2.00 | -18.50 (8), *p*<0.0001 |
| 30 | 0.00±0.00 | 30.00±2.24 | -13.41 (8), *p*<0.0001 |
| 32 | 0.00±0.00 | 25.00±1.58 | -15.81 (8), *p*<0.0001 |
| 34 | 0.00±0.00 | 18.00±1.22 | -14.69 (8), *p*<0.0001 |
| 36 | 0.00±0.00 | 14.00±1.87 | -7.48 (8), *p*<0.0001 |
| 38 | 0.00±0.00 | 11.00±1.87 | -5.87 (8), *p*<0.0001 |
| 40 | 0.00±0.00 | 6.00±1.00 | -6.00 (8), *p*<0.0001 |
| 42 | 0.00±0.00 | 4.00±1.00 | -4.00 (8), *p*=0.004 |
| 44 | 0.00±0.00 | 3.00±1.22 | -2.44 (8), *p*=0.04 |
| 46 | 0.00±0.00 | 1.00±1.00 | -1.00 (8), *p*=0.34 |
| 48 | 0.00±0.00 | 0.00±0.00 | - |

An independent samples *t*-test was performed to compare the mortality data of groups treated with GO and GO-GA NPs (*p* <0.05).
